# Modulation of the Fanconi anemia pathway *via* chemically induced changes in chromatin structure

**DOI:** 10.18632/oncotarget.19470

**Published:** 2017-07-22

**Authors:** David A. Vierra, Jada L. Garzon, Meghan A. Rego, Morganne M. Adroved, Maurizio Mauro, Niall G. Howlett

**Affiliations:** ^1^ Department of Cell and Molecular Biology, University of Rhode Island, Kingston, Rhode Island, U.S.A; ^2^ Addgene, Cambridge, Massachusetts, U.S.A; ^3^ Department of Obstetrics & Gynecology and Women's Health, Albert Einstein College of Medicine, New York, New York, U.S.A

**Keywords:** fanconi anemia, FANCD2, FANCI, monoubiquitination, histone methylation

## Abstract

Fanconi anemia (FA) is a rare disease characterized by congenital defects, bone marrow failure, and atypically early-onset cancers. The FA proteins function cooperatively to repair DNA interstrand crosslinks. A major step in the activation of the pathway is the monoubiquitination of the FANCD2 and FANCI proteins, and their recruitment to chromatin-associated nuclear foci. The regulation and function of FANCD2 and FANCI, however, is poorly understood. In addition, how chromatin state impacts pathway activation is also unknown. In this study, we have examined the influence of chromatin state on the activation of the FA pathway. We describe potent activation of FANCD2 and FANCI monoubiquitination and nuclear foci formation following treatment of cells with the histone methyltransferase inhibitor BRD4770. BRD4770-induced activation of the pathway does not occur *via* the direct induction of DNA damage or *via* the inhibition of the G9a histone methyltransferase, a mechanism previously proposed for this molecule. Instead, we show that BRD4770-inducible FANCD2 and FANCI monoubiquitination and nuclear foci formation may be a consequence of inhibition of the PRC2/EZH2 chromatin-modifying complex. In addition, we show that inhibition of the class I and II histone deacetylases leads to attenuated FANCD2 and FANCI monoubiquitination and nuclear foci formation. Our studies establish that chromatin state is a major determinant of the activation of the FA pathway and suggest an important role for the PRC2/EZH2 complex in the regulation of this critical tumor suppressor pathway.

## INTRODUCTION

All organisms are continuously exposed to endogenous and exogenous DNA damaging agents, including reactive oxygen species and aldehydes from normal metabolic processes and UV irradiation from sunlight. The timely and accurate repair of DNA damage is essential for the maintenance of genome stability and organismal survival. As a consequence, prokaryotic and eukaryotic organisms have evolved complex and highly orchestrated DNA repair pathways to effectively repair damaged DNA. Chromatin represents the higher order macromolecular complex of DNA and histone proteins, and chromatin plasticity has become increasingly recognized as a major determinant of DNA damage recognition, signaling, and repair [1–3]. The nucleosome is the fundamental subunit of chromatin and exhibits plasticity *via* compositional alteration, translational repositioning, and the posttranslational modification of histone tails. Histone tails are subject to a wide variety of posttranslational modifications including acetylation, methylation, phosphorylation, and ubiquitination [4]. Histone acetylation homeostasis is mediated by histone acetyltransferases (HATs), e.g. TIP60/KAT5, and deacetylases (HDACs), e.g. HDAC1 and HDAC2. Underscoring the importance of histone acetylation in DNA repair, key roles for TIP60/KAT5, HDAC1, and HDAC2 in the maintenance of genome stability have been established [5–7].

Fanconi anemia (FA) is a rare autosomal and X-linked genetic disease characterized by congenital defects, bone marrow failure, and increased cancer risk in early adulthood [8]. FA is caused by mutation of any one of 21 genes. The FA proteins function primarily in the repair of DNA interstrand crosslinks (ICLs), lesions that block the replication and transcription machineries, which lead to structural and numerical chromosome aberrations if repaired erroneously [8–11]. A central step in the activation of the FA pathway is the site-specific monoubiquitination of the FANCD2 and FANCI proteins [12–14]. Monoubiquitinated FANCD2 and FANCI localize to discrete sites within chromatin where they are hypothesized to promote the recruitment of several structure-specific endonucleases, including FAN1 (*F*ANCD2*-a*ssociated *n*uclease *1*) and FANCQ/ERCC4 [15–18]. While FANCD2 and FANCI function primarily within chromatin, the contribution of chromatin plasticity, and specifically, the effects of changes in histone tail posttranslational modifications, on their activation and function have yet to be determined. Furthermore, while chromatin remodeling at DNA double-strand breaks (DSBs) has been extensively studied [1–3], very little is known about the role of chromatin remodeling in the context of ICL repair.

In this study we have examined the influence of chromatin structure on the activation of the FA pathway. Specifically, we have examined the effects of histone methyltransferase (HMT), demethylase (HDM), and deacetylase (HDAC) inhibitors on FANCD2 and FANCI monoubiquitination and their assembly into discrete nuclear foci. We describe potent activation of FANCD2 and FANCI monoubiquitination in chromatin, and enhanced FANCD2 and FANCI nuclear foci formation, following cellular exposure to the HMT inhibitor BRD4770. BRD4770-induced activation of the pathway does not appear to occur *via* the direct induction of DNA damage *per se,* or *via* the inhibition of the G9a histone methyltransferase, a mechanism previously proposed for this molecule [19]. In contrast, our results suggest that BRD4770-induced activation of the pathway may be a consequence of inhibition of PRC2 (*P*olycomb *R*epressive *C*omplex *2*) and, specifically, its catalytic HMT EZH2. In addition, we demonstrate that inhibition of class I and II HDACs with trichostatin A (TSA) and vorinostat (SAHA) leads to attenuated ICL-inducible FANCD2 and FANCI monoubiquitination and nuclear foci formation. Our results establish that chromatin plasticity, and in particular the posttranslational modification of histone tails, is a critical determinant in the activation of the FA tumor suppressor pathway.

## RESULTS

### Activation of FANCD2 and FANCI monoubiquitination by the HMTi BRD4770

**Figure 1:**
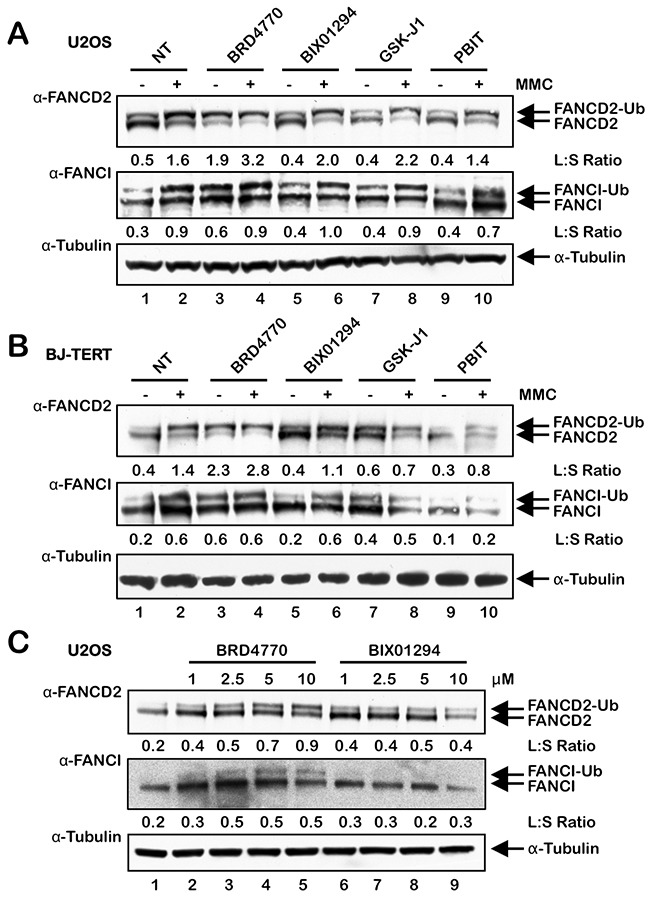
The HMTi BRD4770 induces the monoubiquitination of FANCD2 and FANCI **(A)** and **(B)**, U2OS (A) and BJ-TERT (B) cells were incubated in the absence (NT) or presence of 10 μM BRD4770, 2.5 μM BIX01294, 5 μM GSK-J1 and 1 μM PBIT, with (+) and without (-) 200 nM mitomycin C (MMC) for 24 h. Whole-cell lysates were prepared and immunoblotted with anti-FANCD2, anti-FANCI, and anti-α-Tubulin antibodies. **(C)** U2OS cells were incubated with the indicated concentrations of BRD4770 and BIX01294 for 24 h, and whole-cell lysates were immunoblotted with anti-FANCD2, anti-FANCI, and anti-α-Tubulin antibodies. L:S Ratio, ratio of monoubiquitinated to nonubiquitinated FANCD2 or FANCI.

To explore the effects of global alterations in histone methylation on the activation of the FA pathway, we exposed the transformed osteosarcoma cell line U2OS and the nontransformed telomerase (hTERT)-immortalized line BJ-TERT to the HMT inhibitors (HMTi) BRD4770 and BIX01294 and the HDM inhibitors (HDMi) GSK-J1 and PBIT, and examined FANCD2 and FANCI monoubiquitination. BRD4770 is a *S*-adenosylmethionine (SAM) mimetic and competitive inhibitor of PRC2/EZH2 and G9a [19–21]. BIX01294 is a non-SAM mimetic selective inhibitor of G9a [21]. Treatment with BRD4770 resulted in a marked increase in FANCD2 and FANCI monoubiquitination in both U2OS and BJ-TERT cells, even in the absence of the ICL-inducing agent mitomycin C (MMC) (Figure 1A and 1B, lane 3). Indeed, the ratio of FANCD2-Ub to FANCD2 (L:S ratio) was higher in cells treated with BRD4770 alone than in cells treated with MMC alone (Figure 1A and B, compare lanes 2 and 3). BRD4770-induced activation of FANCD2 monoubiquitination also occurred in a concentration-dependent manner (Figure 1C). In contrast, no major effects on levels of spontaneous or ICL-inducible FANCD2/I monoubiquitination were observed for the other inhibitors tested, other than a slight increase in the FANCD2/I L:S ratios following treatment of BJ-TERT cells with GSK-J1 (Figure 1B).

### BRD4770 promotes FANCD2 chromatin localization and nuclear foci formation

**Figure 2:**
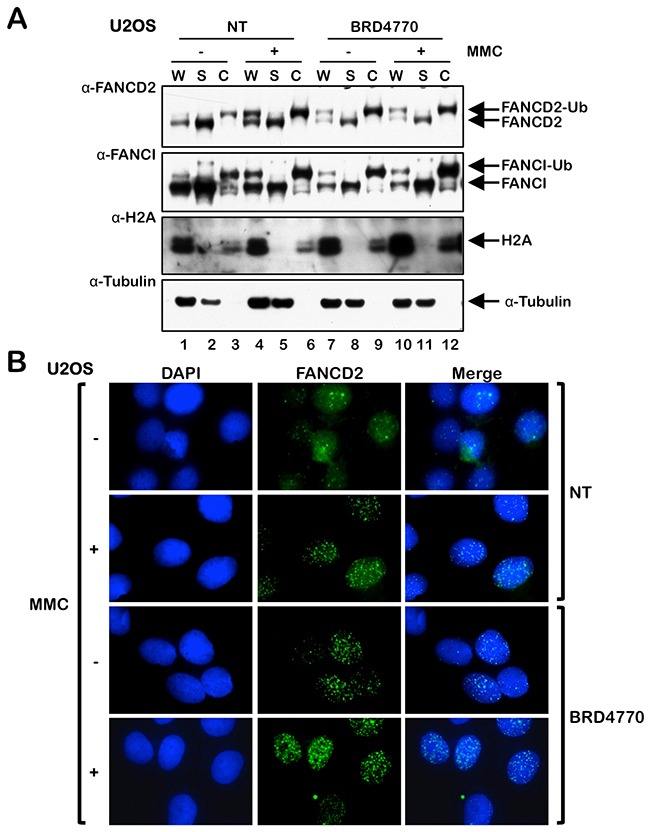
The HMTi BRD4770 induces FANCD2 chromatin localization and nuclear foci formation **(A)** U2OS cells were incubated in the absence (NT) or presence of 10 μM BRD4770 with (+) and without (-) 200 nM mitomycin C (MMC) for 24 h. Whole-cell lysates (W) and soluble nuclear and cytoplasmic (S) and chromatin-enriched (C) fractions were prepared and immunoblotted with anti-FANCD2, anti-FANCI, anti-H2A, and anti-α-Tubulin antibodies. **(B)** U2OS cells were incubated with (+) and without (-) 200 nM MMC in the absence (NT) or presence of 10 μM BRD4770 for 24 h. Cells were fixed and stained with rabbit polyclonal anti-FANCD2 antibody (green) and counterstained with DAPI (blue), and the number of nuclei with >5 FANCD2 foci were scored. At least 300 nuclei were scored for each treatment. Representative immunofluorescence microscopy images are shown.

Next, we examined the effects of BRD4770 treatment on the localization of FANCD2 to chromatin and its assembly into nuclear foci. Cells were incubated in the absence or presence of BRD4770, both with and without MMC, and whole-cell (W), soluble cytoplasmic and nuclear (S), and chromatin-enriched (C) protein lysates were prepared and analyzed by immunoblotting. We observed greatly elevated levels of monoubiquitinated FANCD2 and FANCI in the chromatin fraction of cells treated with BRD4770 alone (Figure 2A, compare lanes 3 and 9). In addition, using immunofluorescence microscopy (IF), we analyzed FANCD2 nuclear foci formation, an indicator of the localization of FANCD2 to sites of damaged DNA in chromatin [12, 22], following treatment with BRD4770 alone and following combined treatment with BRD4770 and MMC. We observed a striking increase in the percentage of nuclei exhibiting greater than 5 FANCD2 foci in both U2OS and BJ-TERT cells following BRD4770 treatment (Figure 2B and Supplementary Figure 1A and 1B). Levels of FANCD2 nuclear foci formation in cells treated with BRD4770 alone were comparable to that observed following MMC treatment (Supplementary Figure 1A and 1B). These results identify BRD4770 as a major inducer of FANCD2 monoubiquitination and nuclear foci formation and strongly suggest that changes in histone methylation status are a critical determinant in the activation of the FA pathway. Consistent with BRD4770 functioning *via* the modification of chromatin structure, we observed a distinct change in the staining pattern of the heterochromatin marker HP1α following BRD4770 treatment (Supplementary Figure 1C).

### BRD4770-inducible activation of the FA pathway does not occur *via* direct induction of DNA damage, increased expression of the FA core complex, or changes in cell cycle progression

**Figure 3:**
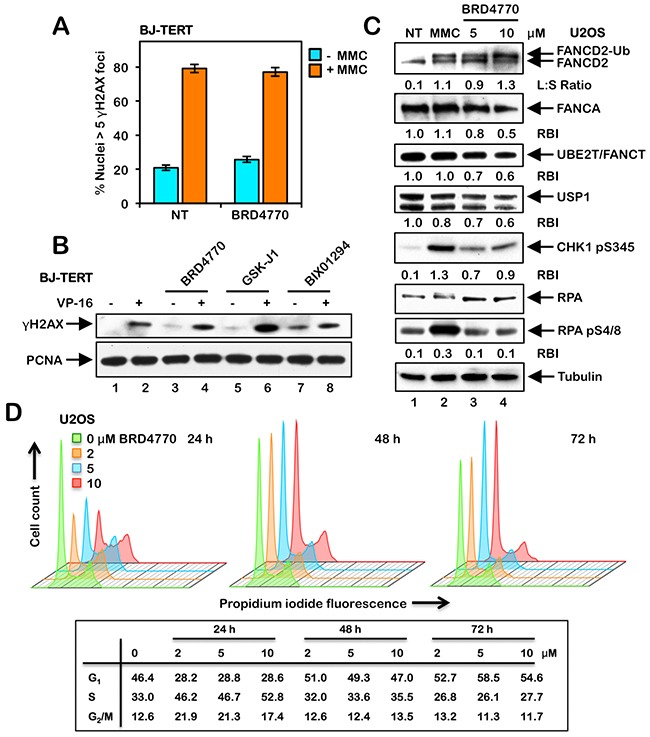
BRD4770-induced activation of the FA pathway does not occur *via* the direct induction of DNA damage or altered cell cycle progression **(A)** BJ-TERT cells were incubated with (+) and without (-) 200 nM mitomycin C (MMC) in the absence (NT) or presence of 10 μM BRD4770 for 24 h. Cells were fixed and stained with mouse monoclonal anti-γH2AX antibody and counterstained with DAPI, and the number of nuclei with >5 γH2AX foci were scored. At least 300 nuclei were scored for each treatment and this experiment was performed three times with similar results. Error bars represent the standard errors of the means from three independent experiments. **(B)** BJ-TERT cells were incubated with (+) and without (-) 0.4 μM etoposide (VP-16), in the absence or presence of 10 μM BRD4770, 5 μM GSK-J1 and 2.5 μM BIX01294, for 24 h. Whole-cell lysates were immunoblotted with anti-γH2AX and anti-PCNA (loading control) antibodies. **(C)** U2OS cells were incubated in the absence (NT) or presence of 200 nM MMC or 5 and 10 μM BRD4770 for 24 h. Whole-cell lysates were prepared and immunoblotted with anti-FANCD2, anti-FANCA, anti-UBE2T, anti-USP1, anti-CHK1 pS345, anti-RPA, anti-RPA pS4/8, and anti-α-Tubulin antibodies. L:S Ratio, ratio of monoubiquitinated to nonubiquitinated FANCD2; RBI, relative band intensity. **(D)** U2OS cells were incubated in the absence or presence of 2, 5, and 10 μM BRD4770 for 24, 48, or 72 h. Cells were fixed in ice-cold ethanol, stained with propidium iodide, and analyzed by flow cytometry. Cell cycle stage distributions were determined using FlowJo v10.2.

**Figure 4:**
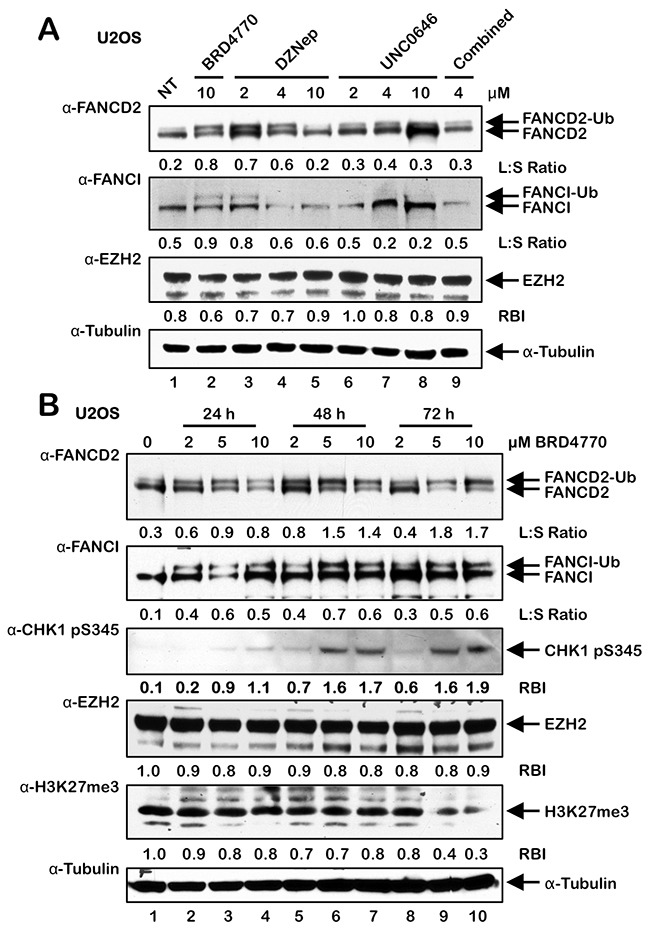
BRD4770-induced activation of the FA pathway may occur *via* inhibition of the PRC2 complex **(A)** U2OS cells were incubated in the absence (NT) or presence of BRD4770, DZNep, UNC0646, and DZNep and UNC0646 combined (4 μM each) for 24 h. Whole-cell lysates were prepared and immunoblotted with anti-FANCD2, anti-FANCI, anti-EZH2, and anti-α-Tubulin antibodies. **(B)** U2OS cells were incubated in the absence or presence of 2, 5, and 10 μM BRD4770 for 24, 48, or 72 h. Whole-cell lysates were prepared and immunoblotted with anti-FANCD2, anti-FANCI, anti-CHK1 pS345, anti-EZH2, anti-H3K27me3, and anti-α-Tubulin antibodies. L:S Ratio, ratio of monoubiquitinated to nonubiquitinated FANCI; or FANCD2 RBI, relative band intensity.

One possible explanation for BRD4770-induced activation of FANCD2 and FANCI monoubiquitination and nuclear foci formation is that BRD4770 induces DNA damage directly. To test this hypothesis, we examined levels of the phosphorylated H2A variant H2AX (γH2AX), a well-established biomarker of DNA DSB formation [23], in cells treated with and without BRD4770. No differences in the number of nuclei exhibiting γH2AX foci were observed between untreated cells and cells treated with BRD4770, both in the absence or presence of MMC (Figure 3A and Supplementary Figure 2). Similar results were observed in both U2OS and BJ-TERT cells (Figure 3A and Supplementary Figure 2). U2OS cells have a highly unstable karyotype, with recurrent breakage-fusion-bridge cycles most likely contributing to the elevated spontaneous levels of γH2AX nuclear foci formation observed in these cells (Supplementary Figure 2). While we observed a faint γH2AX signal for cells treated with BRD4770 alone *via* immunoblotting, this level was markedly lower than that observed following exposure to the topoisomerase type II inhibitor etoposide (VP-16), a well known inducer of DNA DSBs, and no different to that observed following GSK-J1 treatment (Figure 3B). We also examined levels of RPA S4/8 phosphorylation, a marker of single-stranded DNA [24], following BRD4770 exposure. While MMC treatment led to a strong increase in levels of RPA pS4/8, no increase in levels above that of untreated cells was observed for BRD4770 (Figure 3C). We also examined the effects of BRD4770 treatment on levels of the FA core complex proteins FANCA and UBE2T/FANCT. UBE2T/FANCT is the FANCD2 E2 ubiquitin-conjugating enzyme [25]. Levels of both proteins decreased following BRD4770 treatment (Figure 3C). Decreased levels of FANCA were also observed following exposure of HCT116 p53^+/+^ and p53^-/-^ cells to BRD4770 (see Supplementary Figure 3D). Similarly, we observed a reduction in levels of the USP1 de-ubiquitinating enzyme following BRD4770 treatment (Figure 3C). Concomitant reductions in the levels of FANCA, UBE2T/FANCT, and USP1 cannot explain the observed BRD4770-induced FANCD2 and FANCI monoubiquitination and nuclear foci formation. We observed an increase in levels of phosphorylated CHK1 S345 following treatment with BRD4770, albeit to a lesser extent than that observed following MMC treatment (Figure 3C). Finally, we examined the effects of BRD4770 treatment on cell cycle progression. Following exposure to BRD4770 for 24 h, we observed an increase in the percentage of cells in S-phase at all concentrations of BRD4770 examined (Figure 3D). However, following exposure to BRD4770 for 48 and 72 h, where maximal induction of FANCD2 and FANCI monoubiquitination was observed (see Figure 4B), the cell cycle stage profiles did not differ substantially from that of untreated cells (Figure 3D). Taken together, these results argue that BRD4770-induced activation of the FA pathway does not appear to be a consequence of direct induction of DNA damage, alterations in expression of the FANCD2 core monoubiquitination proteins, or major changes to cell cycle progression.

### BRD4770-induced activation of the FA pathway may occur *via* inhibition of the PRC2 complex

BRD4770 is a SAM mimetic and a structural analogue of BIX01338, a non-selective HMT inhibitor with similar IC_50_ values against the G9a and SUV39H1 HMTs [19–21]. Therefore, we next treated U2OS cells with varying concentrations of BIX01338 to determine if we would observe activation of the FA pathway, similar to that observed for BRD4770. Surprisingly, we did not observe any appreciable induction of FANCD2 or FANCI monoubiquitination following treatment with BIX01338 for acute (24 h) (Supplementary Figure 3A) or extended periods (up to 10 d) (results not shown). BRD4770 is a methyl ester analogue of BRD9539, and previous experiments with BRD9539 have shown that it exhibits specificity for G9a and PRC2 (*P*olycomb *R*epressive *C*omplex *2*) [19]. EZH2 is the catalytic HMT of PRC2, and catalyzes the deposition of the transcriptionally repressive mark H3K27me3 [26]. Therefore, to determine if BRD4770-induced activation of FANCD2 monoubiquitination might be a specific consequence of G9a and/or EZH2 inhibition, we treated cells with the DZNep and UNC0646 HMT inhibitors: DZNep treatment has been reported to lead to cellular depletion of EZH2 [27], while UNC0646 is a potent inhibitor of G9a [28]. We observed modest induction of FANCD2 and FANCI monoubiquitination following exposure to 2 and 4 μM DZNep (Figure 4A). Considerable cell toxicity was observed at 10 μM DZNep (results not shown). Under these conditions, we did not observe a decrease in levels of EZH2 expression. However, reduced levels of EZH2 were observed following incubation with DZNep, and BRD4770 to a lesser extent, for 4 and 8 days (Supplementary Figure 3B). In contrast to DZNep, no induction of FANCD2/I monoubiquitination was observed following treatment with UNC0646, and combined DZNep/UNC0646 treatment resulted in considerable cell death (Figure 4A and results not shown). Consistent with these findings, we also observed a modest yet statistically significant increase in FANCD2 nuclear foci formation in cells treated with DZNep and not with UNC0646 (Supplementary Figure 3C). We note that the degree of DZNep-induced FANCD2/I monoubiquitination was experimentally variable, most likely a consequence of its pleiotropic nature. To further explore the potential role of EZH2 and H3K27me3 in BRD4770-inducible FANCD2/I monoubiquitination, we exposed U2OS cells to BRD4770 for 24, 48, and 72 h and examined levels of EZH2 and H3K27me3 (Figure 4B). We observed reductions in levels of H3K27me3 following treatment with higher concentrations of BRD4770 for 72 h, when induction of FANCD2 monoubiquitination was maximal (Figure 4B). Under the same conditions, we did not observe any significant reductions in EZH2 levels (Figure 4B). Similarly, we also observed a modest reduction in levels of H3K27me3 in HCT116 p53^-/-^ cells treated with BRD4770 for 24 h and a more pronounced reduction in HeLa cells treated with BRD4770 for 72 h (Supplementary Figures 3D and 3E). We again observed induction of CHK1 pS345 upon exposure to higher concentrations of BRD4770 for extended periods (Figure 4B).

### EPZ-6438-mediated inhibition of PRC2/EZH2 leads to activation of FANCD2 monoubiquitination

**Figure 5:**
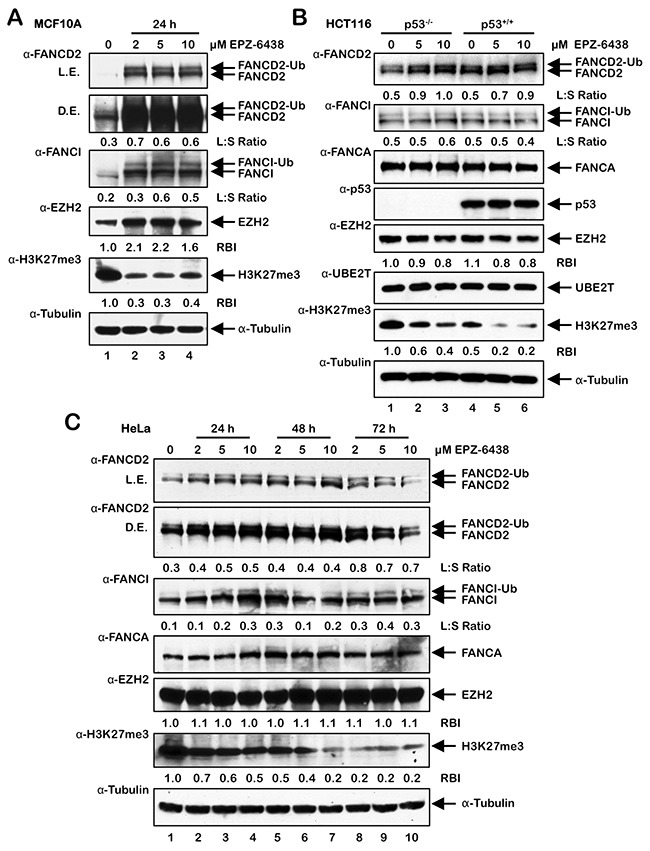
Activation of FANCD2 monoubiquitination following treatment with the EZH2 inhibitor EPZ-6438 **(A-C)** MCF10A (A), HCT116 p53^+/+^ and p53^-/-^ (B), and U2OS (C) cells were treated with the indicated concentrations of the EZH2-specific inhibitor EPZ-6438 for 24 h (A and B) or 24, 48, and 72 h (C). Whole-cell lysates were prepared and immunoblotted with the indicated antibodies. L.E., light exposure; D.E., dark exposure; L:S Ratio, ratio of monoubiquitinated to nonubiquitinated FANCI; or FANCD2 RBI, relative band intensity.

To further analyze the role of PRC2 and EZH2 in the activation of the FA pathway, cells were treated with EPZ-6438, an EZH2-specific inhibitor [29, 30], and FANCD2 and FANCI monoubiquitination and nuclear foci formation were analyzed. In MCF10A cells, a spontaneously-immortalized, nontransformed, mammary epithelial line, EPZ-6438 treatment led to a pronounced increase in FANCD2 and FANCI protein levels, FANCD2 and FANCI monoubiquitination, and FANCD2 nuclear foci formation (Figure 5A and Supplementary Figure 4). Interestingly, EPZ-6438 treatment led to an increase in levels of EZH2, possibly a cellular response to chemical inhibition of EZH2, and an overall reduction in levels of H3K27me3 (Figure 5A). EPZ-6438 treatment also resulted in increased FANCD2 monoubiquitination in isogenic HCT116 p53^+/+^ and p53^-/-^ cells (Figure 5B) and HeLa cells (Figure 5C). In contrast to MCF10A, HeLa, and HCT116, we did not detect increased FANCD2 monoubiquitination in U2OS cells treated with EPZ-6438 (results not shown). Taken together, our results suggest that BRD4770-induced activation of the FA pathway may occur *via* inhibition of the PRC2 complex, and specifically EZH2 HMT activity, and a consequent decrease in levels of H3K27me3.

### Inhibition of class I and II HDACs attenuates activation of the FA pathway

**Figure 6:**
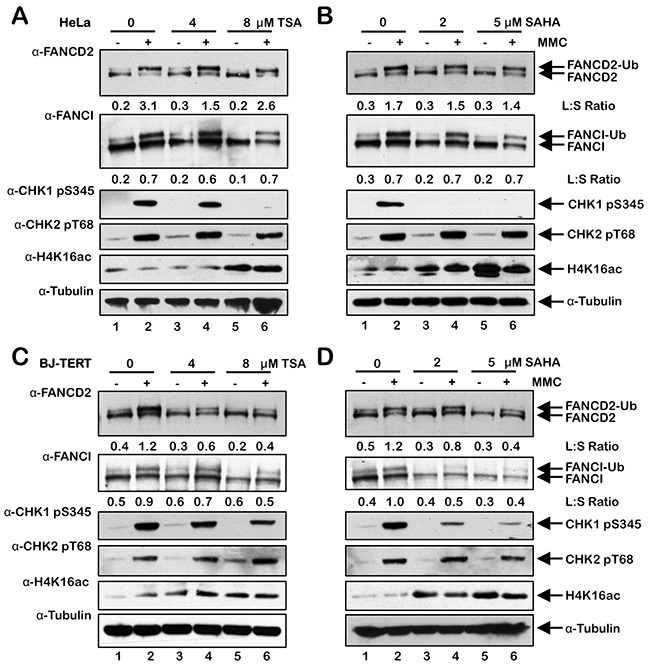
Inhibition of class I and II HDACs attenuates FANCD2 and FANCI monoubiquitination in BJ-TERT cells **(A)** and **(B)**, HeLa cells were pre-treated with the indicated concentrations of trichostatin A (TSA) (A) or vorinostat (SAHA) (B) for 4 h, followed by co-incubation with (+) and without (-) 200 nM MMC for a further 20 h. Whole-cell lysates were prepared and immunoblotted with anti-FANCD2, anti-FANCI, anti-CHK1 pS345, anti-CHK2 pT68, anti-H4K16ac, and anti-α-Tubulin antibodies. **(C)** and **(D)**, BJ-TERT cells were treated identically to that described for HeLa cells above. L:S Ratio, ratio of monoubiquitinated to nonubiquitinated FANCD2 or FANCI.

**Figure 7:**
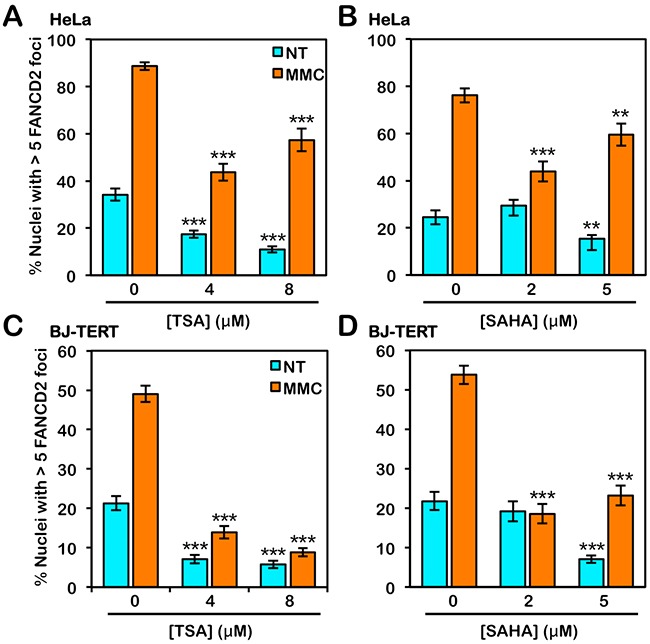
Inhibition of class I and II HDACs attenuates FANCD2 nuclear foci formation **(A)** and **(B)**, HeLa cells were treated with the indicated concentrations of trichostatin A (TSA) (A) or vorinostat (SAHA) (B) in the absence (NT) or presence (MMC) of 200 nM mitomycin C (MMC) for 24 h. Cells were fixed and stained with rabbit polyclonal anti-FANCD2 antibody and counterstained with DAPI, and the number of nuclei with >5 FANCD2 foci were scored. *, *P* < 0.05; **, *P* < 0.01; ***, *P* < 0.001, with comparison to untreated cells. **(C)** and **(D)**, BJ-TERT cells were treated identically to that described for HeLa cells above. At least 300 nuclei were scored for each treatment and these experiments were performed three times with similar results. Error bars represent the standard errors of the means from three independent experiments.

To investigate the influence of histone acetylation state on the activation of the FA pathway, we next examined the effects of the class I and II HDAC inhibitors trichostatin A (TSA) and vorinostat (SAHA) on FANCD2 and FANCI monoubiquitination and nuclear foci formation. Interestingly, for HeLa cells, we did not observe any appreciable differences in the levels of spontaneous or ICL-inducible FANCD2 or FANCI monoubiquitination when cells were treated with TSA or SAHA (Figure 6A and 6B). In contrast, when BJ-TERT cells were treated with TSA or SAHA, we observed a marked reduction in the levels of ICL-inducible FANCD2 and FANCI monoubiquitination (Figure 6C and 6D). TSA and SAHA treatment led to a reduction in ICL-inducible CHK1 S345 phosphorylation in both lines examined (Figure 6). In contrast, incubation with TSA and SAHA did not lead to any observable changes in levels of ICL-inducible CHK2 pT68 (Figure 6). We observed a concentration-dependent increase in the levels of H4K16ac following treatment with TSA and SAHA confirming their inhibition of histone deacetylation (Figure 6). We also observed a reduction in spontaneous and ICL-inducible FANCD2 nuclear foci formation in both HeLa and BJ-TERT cells treated with TSA or SAHA (Figure 7A–7D). Consistent with our immunoblotting results, this effect was particularly striking for BJ-TERT cells with a lesser effect observed for HeLa cells (Figure 7A–7D).

**Figure 8:**
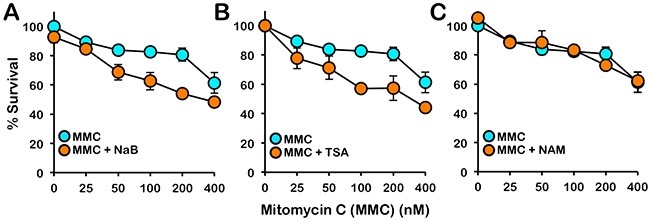
Inhibition of class I and II HDACs sensitizes cells to the cytotoxic effects of mitomycin C BJ-TERT cells were exposed to the indicated concentrations of mitomycin C (MMC) in the absence or presence of **(A)** 5 mM sodium butyrate (NaB), **(B)** 331 nM trichostatin A (TSA), or **(C)** 10 mM nicotinamide (NAM) and cellular proliferation was determined using the CellTiter 96® AQ_ueous_ One Solution Cell Proliferation (MTS) assay. Error bars represent the standard errors of the means from three independent experiments.

The FA pathway is a major determinant of cellular sensitivity to ICL-inducing agents. Therefore we next examined if treatment with HDAC I and II inhibitors would sensitize cells to the cytotoxic effects of MMC. Indeed, even at the low concentrations of inhibitors examined, treatment with the HDAC I and II inhibitors sodium butyrate (NaB) and TSA sensitized BJ-TERT cells to the cytotoxic effects of MMC over a range of MMC concentrations (Figure 8A and 8B). In contrast, treatment with nicotinamide (NAM), an inhibitor of the NAD+ (nicotinamide adenine dinucleotide)-dependent sirtuin (class III) family of HDACs, did not impact cellular sensitivity to MMC (Figure 8C). Taken together, our results indicate that HDAC1 and HDAC2 positively regulate activation of the FA pathway and that cellular sensitivity to ICL-inducing agents, which are widely used in cancer chemotherapy, may be increased *via* HDAC1/2 inhibition.

## DISCUSSION

In this study, we have established that chromatin state is an important determinant in the activation of the FA pathway. Specifically, we have established that treatment with the HMTi BRD4770 and inhibition of the class I and II HDACs strongly impacts activation of the FA pathway. BRD4770 was recently discovered in a focused screen of a 2-substituted benzimidazole library [19]. While in *in vitro* biochemical assays, BRD9539, the carboxylic acid derivative of BRD4770, effectively inhibited both G9a and PRC2/EZH2 in a concentration-dependent manner, these studies concluded that BRD4770 functions primarily *via* the inhibition of G9a [19]. However, in our experiments, while BRD4770 robustly promoted FA pathway activation, several G9a-specific inhibitors - including BIX01294 and UNC0646 - failed to promote FA pathway activation. Our results suggest that BRD4770 does not act primarily *via* G9a inhibition, and that G9a does not play a significant role in the regulation of the FA pathway. Instead, our findings suggest that BRD4770 may exert its cellular effects, at least in part, *via* the inhibition of PRC2/EZH2, and that PRC2/EZH2 may play an important role in the regulation of the activation of the FA pathway. Several findings support this model: Exposure to BRD4770 resulted in reduced levels of H3K27me3 - the transcriptionally repressive mark deposited by PRC2/EZH2 [26] - in several cell models. Modest activation of FANCD2 monoubiquitination and nuclear foci formation was observed following treatment with the PRC2/EZH2 inhibitor DZNep [27]. However, while DZNep was initially reported to be selective for PRC2/EZH2 [27], as a *S*-adenosylhomocysteine hydrolase inhibitor, DZNep can also affect global histone methylation patterns [31]. This led us to examine the effects of inhibition of EZH2 with the SAM-competitive inhibitor EPZ-6438. EPZ-6438 is a potent and selective EZH2 inhibitor with an inhibition constant (*K*_i_) of 2.5 nM. EPZ-6438 is 35-fold and >4,500-fold more selective for EZH2 than EZH1 and 14 other HMTs examined [32]. In four out of five lines examined in this study, EPZ-6438 treatment led to an increase in levels of FANCD2 monoubiquitination. The mechanism(s) by which inhibition of PRC2/EZH2 and decreased global levels of H3K27me3 would lead to activation of the FA pathway remain to be clearly elucidated. Recent studies in the silkworm *Bombyx mori* have shown that PRC2-mediated H3K27me3 increases following exposure to UV irradiation [33]. One possibility is that, upon exposure to DNA damaging agents, transcription may need to be halted at loci that have incurred DNA damage. An inability to catalyze H3K27me3 and arrest transcription could lead to the formation of co-transcriptional RNA-DNA hybrids (R-loops). An important role for the FA proteins in the repair of R-loops has recently been established [34, 35]. However, our γH2AX and RPA pS4/8 results strongly suggest a DNA damage-independent mode of action for BRD4770. An alternative hypothesis is that BRD4770 treatment - possibly *via* both G9a and PRC2/EZH2 inhibition - leads to the general establishment of a transcriptionally permissive chromatin state, which leads to the recruitment of factors that promote homologous recombination (HR) DNA repair, such as FANCD2 and FANCI [13, 36]. Consistent with this hypothesis, Aymard *et al* have recently shown that HR factors are enriched at transcriptionally active chromatin [37]. It is important to note, however, that BRD4770-induced activation of the FA pathway most likely does not occur solely *via* the inhibition of PRC2/EZH2: BRD4770 induces FANCD2/I monoubiquitination more robustly than the EZH2-specific inhibitor EPZ-6438, and EPZ-6438 treatment results in more pronounced decreases in levels of H3K27me3. Therefore, other mechanisms are likely to contribute to the observed effects of BRD4770. In our study, we also detected increased phosphorylation of CHK1 S345 following BRD4770 exposure, indicating activation of the ATR-CHK1 checkpoint-signaling pathway. Numerous studies have indicated an important role for this pathway in signaling aberrant changes in chromatin structure [38–40]. One possibility is that BRD4770-induced changes in chromatin state lead to activation of ATR, phosphorylation of CHK1 and monoubiquitination of FANCD2, leading to activation of the intra-S-phase checkpoint. These findings are consistent with a large body of evidence linking the ATR-CHK1 signaling pathway to the activation of the FA pathway [41–44]. However, while BRD4770 treatment for 24 h did lead to an accumulation of cells in S-phase, exposure to BRD4770 for longer periods - when levels of phosphorylated CHK1 S345 and monoubiquitinated FANCD2 were maximal - did not appear to result in an overt S-phase arrest. Further experiments will be required to determine the relationship between changes in chromatin state, activation of the ATR-CHK1-FANCD2 axis and maintenance of the intra-S-phase checkpoint.

Here, we have also established that chemical inhibition of class I and II HDACs leads to attenuation of FANCD2 and FANCI monoubiquitination in BJ-TERT cells, with a less pronounced effect in HeLa cells, and significant reductions in FANCD2 nuclear foci formation in both HeLa and BJ-TERT cells. FANCD2/I monoubiquitination and nuclear foci are not strictly coupled: *Usp1^-/-^* murine cells exhibit increased Fancd2 monoubiquitination in the absence of nuclear foci formation [45]. Thus, under the conditions examined, our findings point to an important role for histone deacetylation in facilitating the efficient activation of the FA pathway. Previous studies in the budding yeast *S. cerevisiae*, have shown that valproic acid (VPA), a class I and II HDACi, inhibits Mec1 (orthologue of human ATR) signaling [46]. This is consistent with our observation of reduced ICL-inducible CHK1 S345 phosphorylation following HDAC inhibition with SAHA and TSA. Taken together with our BRD4770 findings, our results indicate that the ATR-CHK1 signaling pathway responds to changes in chromatin structure, and that phosphorylation and activation of CHK1 correlates with monoubiquitination and activation of FANCD2.

Several studies have established that, during the very early stages of DSB repair - seconds to minutes - a transient repressive chromatin state is first established, characterized by the spreading of HP1 and H3K9me2/3 [47]. The multisubunit NuRD repressor complex is rapidly recruited to sites of DNA damage where it promotes histone deacetylation and chromatin remodeling [48–50]. This may be necessary to restrict transcription in the immediate vicinity of the damaged site and to promote the recruitment of repair factors [47]. A failure to rapidly establish this transient repressive chromatin state would be predicted to lead to inefficient activation of DNA repair pathways, as we have observed following ICL treatment and HDAC inhibition. In contrast, a failure to catalyze H3K27me3 upon exposure to BRD4770 or EPZ-6438 may lead to persistent, constitutively open/relaxed chromatin, which in turn may promote the inadvertent activation of repair mechanisms, as previously shown [51].

In summary, our results establish that chromatin state is an important determinant of the activation of the FA pathway. Our findings also suggest that combination chemotherapy comprising ICL-inducing agents and HDAC inhibitors may be an effective strategy for certain cancers. Recent studies have identified three functional modules within the FA core complex: the FANCB-FANCL-FAAP100 module, which provides the essential monoubiquitination catalytic activity, and the FANCA-FANCG-FAAP20 and FANCC-FANCE-FANCF modules, which are thought to promote the recruitment of the core complex to chromatin [52, 53]. The majority of FA patients harbor mutations in the *FANCA* and *FANCG* genes, and FANCD2/I monoubiquitination is defective in >95% of FA patients [54]. Based on our findings, it is conceivable that the chromatin recruitment of the monoubiquitination catalytic module could be promoted *via* chemical modification of chromatin state, raising the prospect of epigenetics-based therapeutic approaches for certain FA complementation groups.

## MATERIALS AND METHODS

### Cell culture

The osteosarcoma cell line U2OS, the cervical carcinoma cell line HeLa, and the hTERT-immortalized BJ-TERT cells were grown in DMEM supplemented with 15% v/v fetal bovine serum, 2 mM L-glutamine, 50 U/mL penicillin, and 50 μg/mL streptomycin. HCT116 p53^+/+^ and p53^-/-^ cells were grown in McCoy's 5A medium containing the same supplements [55]. MCF10A mammary epithelial cells were grown in DMEM F12 supplemented with 5% v/v horse serum, 20 ng/ml epidermal growth factor, 0.5 mg/ml hydrocortisone, 100 ng/ml cholera toxin, 10 μg/ml insulin, 2 mM L-glutamine, 50 U/mL penicillin, and 50 μg/mL streptomycin.

### Chemicals

The structures of all chemicals used in this study are shown in Supplementary Figure 5. The following chemicals were used: BRD4770 (histone methyltransferase inhibitor VI) (C_25_H_23_N_3_O_3_) (382194; EMD Millipore), BIX01294 (C_28_H_38_N_6_O_2_.3HCl.xH_2_O) (B9311; Sigma), GSK-J1 (histone lysine demethylase inhibitor VII) (C_22_H_23_N_5_O_2_) (420204; EMD Millipore), PBIT (histone lysine demethylase inhibitor IX) (C_14_H_11_NOS) (505299; EMD Millipore), UNC0646 (C_36_H_59_N_7_O_2_) (SML0633; Sigma), BIX01338 (C_32_H_24_F_3_N_3_O_6_.xH_2_O) (B5313; Sigma), DZNep (3-Deazaneplanocin A) (C_12_H_14_N_4_O_3_) (13828; Cayman Chemical), EPZ-6438 (Tazemetostat) (C_34_H_44_N_4_O_4_) (S7128; Selleckchem), SAHA (Vorinostat) (C_14_H_20_N_2_O_3_) (SML0061; Sigma), Trichostatin A (TSA) (C_17_H_22_N_2_O_3_) (T1952; Sigma), Mitomycin C (MMC) (C_15_H_18_N_4_O_5_) (BP25312; Fisher Scientific), and Etoposide (VP-16) (C_29_H_32_O_13_) (E1383; Sigma).

### Immunoblotting and antibodies

For immunoblotting analysis, cell pellets were washed in PBS and lysed in 2% w/v SDS, 50 mM Tris-HCl, 10 mM EDTA. Proteins were resolved on NuPage 3-8% w/v Tris-Acetate or 4-12% w/v Bis-Tris gels (Invitrogen) and transferred to polyvinylidene difluoride (PVDF) membranes. The following antibodies were used: mouse monoclonal sera against γH2AX (05-636; Millipore), HDAC1 (5356; Cell Signaling), HDAC2 (5113; Cell Signaling), HP1α (05-689; Millipore), PCNA (sc-56; Santa Cruz Biotechnology), RPA (NA18; Calbiochem), and α-tubulin (MS-581-PO; Neomarkers), rabbit monoclonal serum against CHK1 pS345 (2348; Cell Signaling), and rabbit polyclonal sera against CHK2 pT68 (2661; Cell Signaling), EZH2 (5246S; Cell Signaling), FANCA (ABP6201; Cascade), FANCD2 (NB100-182; Novus Biologicals), FANCI (A301-254A; Bethyl Laboratories), H2A (07-146; Millipore), H3K27me3 (9733P; Cell Signaling), H4K16ac (07-329; Millipore), RPA pS4/8 (A300-245A; Bethyl), USP1 (a kind gift from Tony T. Huang, New York University), and UBE2T (A301-874A; Bethyl).

### Immunofluorescence microscopy

For immunofluorescence microscopy (IF) analysis, cells were seeded in 4-well tissue culture slides (BD Falcon) in the presence or absence of drug(s) for 24 h. Soluble cellular proteins were pre-permeabilized with 0.3% v/v Triton X-100 and cells were fixed in 4% w/v paraformaldehyde and 2% w/v sucrose at 4°C followed by permeabilization in 0.3% v/v Triton X-100 in PBS. Fixed cells were blocked for 30 minutes in antibody dilution buffer (5% v/v goat serum, 0.1% v/v NP-40, in PBS) and incubated with primary antibody for 1 h. Cells were washed three times in PBS, as well as permeabilization buffer, and incubated for 30 min at room temperature with an Alexa Fluor 488-conjugated secondary antibody and the slides were counterstained and mounted in vectashield plus 4’6-diamidine-2-phenylindole dihydrochloride (DAPI) (Vector Laboratories). Nuclear foci were scored using a Zeiss AxioImager.A1 upright epifluorescence microscope with AxioVision LE 4.6 image acquisition software. Primary antibodies used for IF were anti-FANCD2 (NB100-182; Novus Biologicals), anti-FANCI (A300-212A; Bethyl Laboratories), and anti-γH2AX (05-636; Millipore). Statistical significance was determined using paired two-tailed Student's *t*-test analysis.

### Chromatin fractionation

Cells were plated at density of 3 × 10^6^ cells in 15 cm^2^ dishes. The following day, cells were treated with 200 nM MMC for 24 h. Cells were harvested and resuspended in ice-cold PBS. A portion of the pellet was retained as a whole cell lysate (W). The remaining pellet was lysed on ice in cytoskeletal buffer (CSK) (300 mM Sucrose, 100 mM NaCl, 3 mM MgCl_2_, 0.5% v/v Triton-X-100, 1 mM EGTA, 10 mM PIPES, pH 6.8). The supernatant, containing soluble cytoplasmic and nuclear proteins, was collected as the soluble fraction (S). The remaining pellet, containing chromatin-associated and nuclear insoluble proteins (C), and the whole-cell lysate pellet, were lysed in 2% SDS lysis buffer with sonication for 10 s at 10% amplitude using a Fisher Scientific Model 500 Ultrasonic Dismembrator.

### Cell proliferation assay

Cells were plated at a density of 10,000 cells/well in 96-well dishes, incubated in the absence or presence of drug(s) for 48 h. CellTiter 96® AQ_ueous_ One Solution Reagent (MTS) (Promega) was added directly to the wells, incubated for a further 2 h, and the absorbance at 490 nm was measured using a 96-well Bio-Rad 680 microplate reader.

### Cell-cycle analysis

Cells were plated at a density of 1×10^6^ cells in 10 cm^2^ dishes. The following day, cells were incubated in the absence or presence of 2, 5, and 10 μM BRD4770 for 24, 48, and 72 h. Cells were resuspended in 0.1 mL PBS and fixed by adding 1 mL ice-cold methanol. Cells were washed in PBS and incubated in 50 μg/mL propidium iodide (PI) (Sigma) and 30 U/mL RNase A for 10 min at 37°C, followed by analysis using a BD FACSVerse flow cytometer. The percentages of cells in G1, S, and G2/M were determined by analyzing PI histograms with FlowJo V10.2 software.

## SUPPLEMENTARY MATERIALS FIGURES AND TABLES



## Abbreviations

HAT: histone acetyltransferase
HDAC: histone deacetylase
HDM: histone demethylase
HMT: histone methyltransferase
ICL: DNA interstrand crosslink
MMC: mitomycin C
NT: no treatment
VP-16: etoposide.
