# Supplementary material for: Modulation of the Fanconi anemia pathway via chemically induced changes in chromatin structure
Source: Oncotarget. 2017 Jul 22;8(44):76443–57. doi: 10.18632/oncotarget.19470 (PMC5652718; doi:10.18632/oncotarget.19470)
Supplement: Supplementary file 1 [file oncotarget-08-76443-s001.pdf]

Modulation of the fanconi anemia pathway *via* chemically induced changes in chromatin structure

SUPPLEMENTARY MATERIALS

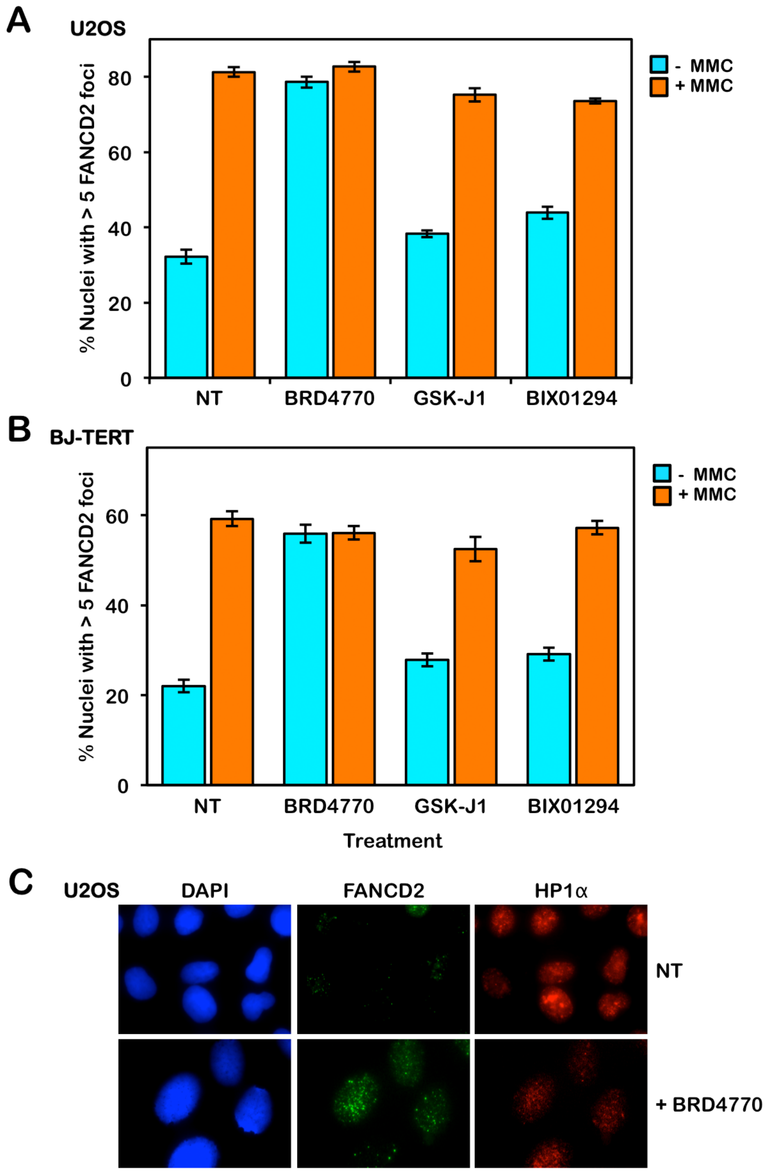

Supplementry Figure 1: The HMTi BRD4770 induces FANCD2 nuclear foci formation. (A) U2OS and **(B)** BJ-TERT cells were incubated in the absence (NT) or presence of 10 µM BRD4770, 5 µM GSK-J1, and 2.5 µM BIX01294, with (+) and without (-) 200 nM MMC for 24 h. Cells were fixed and stained with rabbit polyclonal anti-FANCD2 antibody (green) and counterstained with DAPI (blue), and the number of nuclei with >5 FANCD2 foci were scored. At least 300 nuclei were scored for each treatment and this experiment was performed at least three times with similar results. Error bars represent the standard errors of the means from three independent experiments. **(C)** BRD4770 treatment leads to changes in the staining pattern of the heterochromatin marker HP1α. U2OS cells were incubated in the absence (NT) or presence of 10 µM BRD4770 for 24 h. Cells were fixed and stained with rabbit polyclonal anti-FANCD2 antibody (green), mouse monoclonal anti-HP1α antibody (red), and counterstained with DAPI (blue). Representative images from two independent experiments are shown.

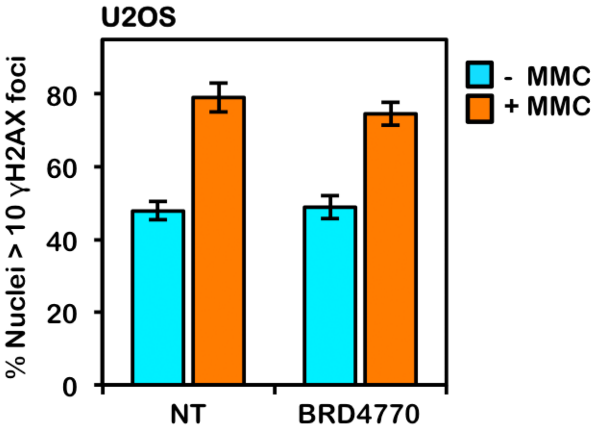

Supplementry Figure 2: BRD4770-induced activation of the FA pathway does not occur *via* the direct induction of DNA damage. U2OS cells were incubated with (+) and without (-) 200 nM mitomycin C (MMC) in the absence (NT) or presence of 10 μM BRD4770 for 24 h. Cells were fixed and stained with mouse monoclonal anti-γH2AX antibody and counterstained with DAPI, and the number of nuclei with >10 γH2AX foci were scored. At least 300 nuclei were scored for each treatment and this experiment was performed three times with similar results. Error bars represent the standard errors of the means from three independent experiments.

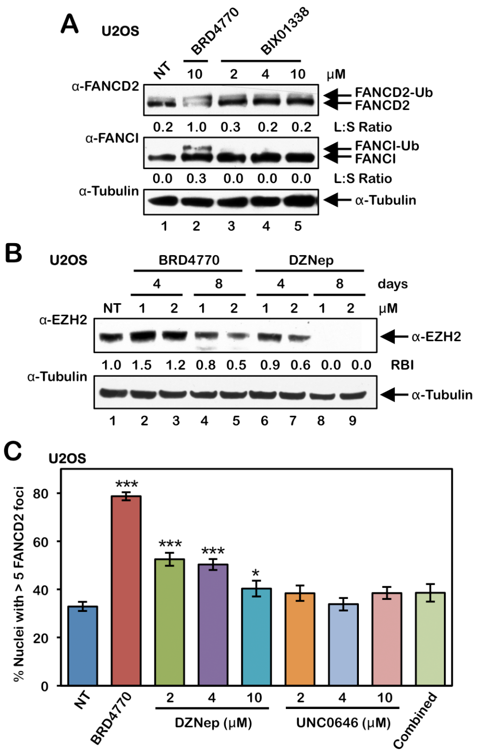

Supplementry Figure 3: BRD4770-induced activation of the FA pathway may occur *via* inhibition of the PRC2 complex. **(A)** U2OS cells were incubated in the absence (NT) or presence of the indicated concentrations of BRD4770 or BIX01338 for 24 h. Whole-cell lysates were prepared and immunoblotted with anti-FANCD2, anti-FANCI, and anti-α-Tubulin antibodies. **(B)** U2OS cells were incubated with the indicated concentrations of BRD4770 and DZNep for 4 and 8 days. Whole-cell lysates were prepared and immunoblotted with anti-EZH2 and anti-α-Tubulin antibodies. L:S Ratio, ratio of monoubiquitinated to nonubiquitinated FANCD2; or FANCI RBI, relative band intensity. **(C)** U2OS cells were incubated with 10 µM BRD4770 or the indicated concentrations of DZNep or UNC0646 for 24 h. Cells were fixed and stained with rabbit polyclonal anti-FANCD2 antibody and counterstained with DAPI, and the number of nuclei with >5 FANCD2 foci were scored. At least 300 nuclei were scored for each treatment and this experiment was performed three times with similar results. Error bars represent the standard errors of the means from three independent experiments. *, $P < 0.05$; **, $P < 0.01$; ***, $P < 0.001$, with comparison to untreated cells. (*Continued*)

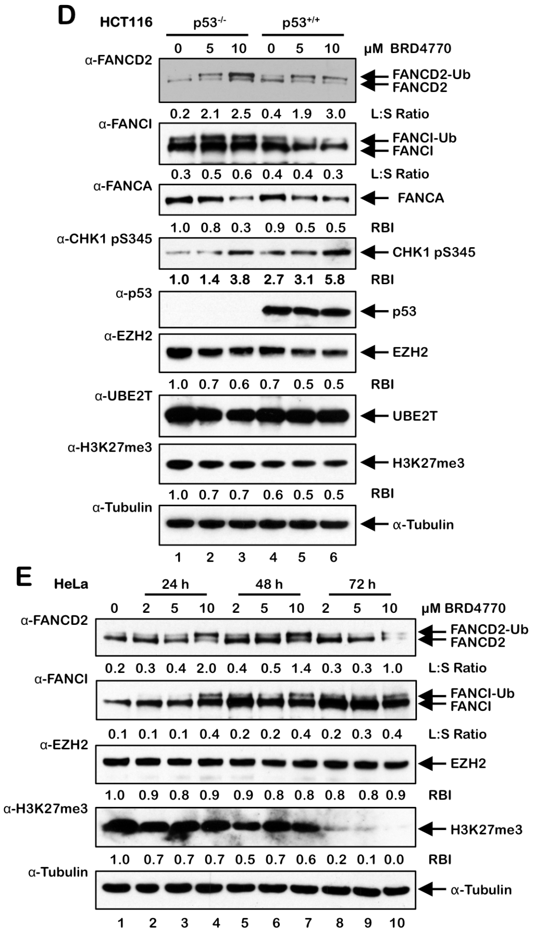

Supplementry Figure 3: BRD4770-induced activation of the FA pathway may occur *via* inhibition of the PRC2 complex. (*Continued*) (**D** and **E**) HCT116 $p53^{+/+}$ and $p53^{-/-}$ (D) and HeLa (E) cells were incubated in the absence or presence of the indicated concentrations of BRD4770 for 24 h (D) or 24, 48, or 72 h (E). Whole-cell lysates were prepared and immunoblotted with the indicated antibodies. L:S Ratio, ratio of monoubiquitinated to nonubiquitinated FANCD2; or FANCI RBI, relative band intensity.

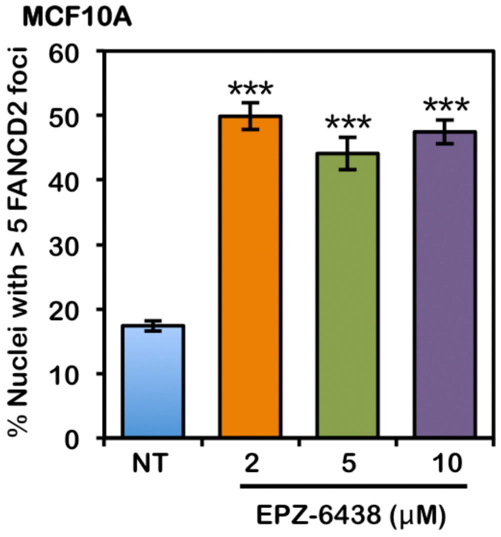

Supplementry Figure 4: Activation of FANCD2 monoubiquitination following treatment with the EZH2 inhibitor EPZ-6438. MCF10A cells were treated with the indicated concentrations of the EZH2-specific inhibitor EPZ-6438 for 24 h. Cells were fixed and stained with rabbit polyclonal anti-FANCD2 antibody and counterstained with DAPI, and the number of nuclei with >5 FANCD2 foci were scored. At least 300 nuclei were scored for each treatment and this experiment was performed twice with similar results. Error bars represent the standard errors of the means from two independent experiments. *** $P < 0.001$, with comparison to untreated cells.

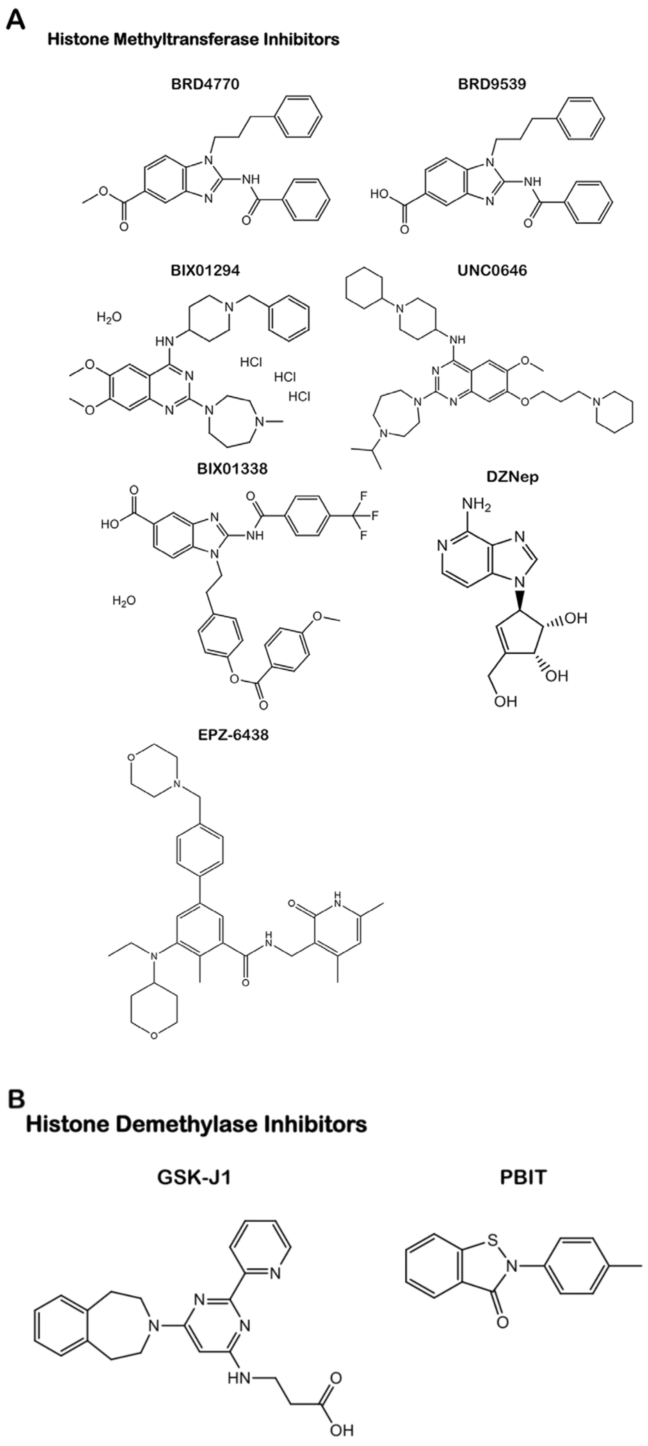

Supplementry Figure 5: Chemical structures of the inhibitors and DNA damaging agents used throughout this study. **(A)** Histone methyltransferase inhibitors, **(B)** histone demethylase inhibitors. (*Continued*)

C **Histone Deacetylase Inhibitors**

Vorinostat (SAHA)

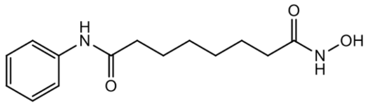

Trichostatin A (TSA)

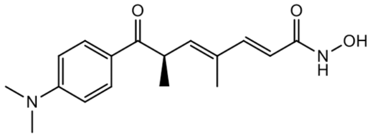

Sodium Buryrate (NaB)

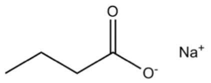

Nicotinamide (NAM)

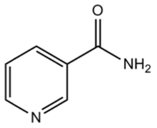

D **Miscellaneous**

Etoposide (VP-16)

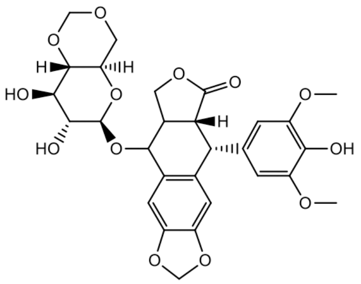

Mitomycin C (MMC)

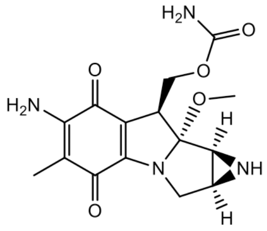

Supplementry Figure 5: (*Continued*)Chemical structures of the inhibitors and DNA damaging agents used throughout this study. **(C)** histone deacetylase inhibitors, and **(D)** miscellaneous DNA damaging agents.
